# Repair of cerebrospinal fluid leak during posterior thoracolumbar surgery using paraspinal muscle flap combined with fat graft

**DOI:** 10.3389/fsurg.2022.969954

**Published:** 2022-10-10

**Authors:** Xianda Gao, Peiyu Du, Jiaxin Xu, Jiayuan Sun, Wenyuan Ding, Da-Long Yang

**Affiliations:** Third Hospital of Hebei Medical University, Shijiazhuang, China

**Keywords:** cerebrospinal fluid leakage, posterior approach, thoracolumbar surgery, muscle flap, fat graft

## Abstract

**Objective:**

This study aimed to propose a novel surgical method *via* combination of fat graft and paraspinal muscle flap, in order to treat cerebrospinal fluid (CSF) leak during posterior thoracolumbar surgery. The clinical outcomes were also evaluated.

**Methods:**

Data of a total of 71 patients who were diagnosed with intraoperative incidental durotomy and CSF leak after posterior thoracolumbar surgery in our hospital form January 2019 to January 2021 were retrospectively collected and analyzed. Among them, 34 and 37 patients were assigned into conventional suturing (CS) group and fat graft and paraspinal muscle flap (FPM) group, respectively. Patients’ demographic and clinical data were compared between the two groups.

**Results:**

The average drainage tube time in the FPM group was 3.89 ± 1.17 days, which was shorter than that in the CS group (5.12 ± 1.56, *P* < 0.001). The drainage volume in the FPM group (281.08 ± 284.76 ml) was also smaller than that in the CS group (859.70 ± 553.11 ml, *P* < 0.001). Besides, 15 (44.11%) patients in the CS group complained of postural headache, which was more than that in the FPM group (7 patients, 18.91%). There was a statistically significant difference in postoperative visual analogue scale (VAS) score between the two groups (*P* = 0.013). Two patients underwent revision surgery resulting from incision nonunion and delayed meningeal cyst.

**Conclusion:**

Fat graft combined with paraspinal muscle flap showed to be an effective method to repair CSF leak during posterior thoracolumbar surgery. The proposed method significantly reduced postoperative drainage tube time and postoperative drainage volume. It also decreased the incidence and the degree of postural headache. The proposed method showed satisfactory clinical outcomes, and it is worthy of promotion.

## Background

Cerebrospinal fluid (CSF) leak after posterior thoracolumbar surgery is mainly caused by incidental durotomy intraoperatively, especially in the occasion of dural adhesion with surrounding tissue or abnormal ossification (1–4). As a common complication, the incidence rate of CSF leak ranges from 1% to 17.4% (4–7). The effective treatment for CSF leak is dural suturing during surgery, however, anterior and a part of lateral dural rupture are difficult to suture. In addition, intraoperative suturing does not always fully prevent outflow of CSF (8, 9). Persistent postoperative CSF leak could lead to various low intracranial pressure symptoms, such as headache, dizziness, nausea, and vomiting (1, 4). Moreover, wound complications arose with the continuous outflow of CSF, such as nonunion, infection, and pseudomeningocele formation. Severe complications caused by CSF leak require revision surgery (3). CSF leak negatively affects clinical outcomes and patient satisfaction. Although different methods have been proposed to repair damaged dura mater and a variety of biological materials were used to block CSF leak, spinal surgeons still encounter numerous multifarious problems caused by CSF leak. The present study aimed to propose a combined method using fat graft and paraspinal muscle flap, in order to treat CSF leak after posterior thoracolumbar surgery and to evaluate the clinical outcomes. This novel method may assist clinicians to reduce the influence of CSF leak.

## Study subjects and methods

### Study subjects

Data of a total of 71 patients who were diagnosed with intraoperative incidental durotomy and CSF leak after posterior thoracolumbar surgery at the Third Hospital of Hebei Medical University (Shijiazhuang, China) form January 2019 to January 2021 were retrospectively collected and analyzed. The inclusion criteria were as follows: (1) patients who underwent thoracolumbar spine surgery; (2) patients who underwent posterior surgery only; (3) intraoperative CSF leak caused by incidental durotomy; (4) patients who aged >18 years old; (5) follow-up of at least 12-month. The exclusion criteria were as follows: (1) intraspinal tumors; (2) spinal infectious diseases; (3) a history of undergoing surgery involving spine; (4) intraoperative application of special biomaterials (e.g., an artificial dura mater); (5) incomplete clinical data.

There were 25 (35.21%) men and 46 (64.79%) women who were enrolled in the present study, and their average age was 51.64 ± 11.92 years old. The average follow-up time was 15.10 ± 4.15 months. The patients were operated by the same surgeon. Conventional suturing was used in 34 patients, and they were assigned into conventional suturing (CS) group. Fat graft and paraspinal muscle flap were used in other 37 patients to reduce CSF leak, and they were assigned into fat graft and paraspinal muscle flap (FPM) group. In addition, patients’ basic characteristics, including body mass index (BMI), medical comorbidities (hypertension, diabetes, cardiac disease, cerebrovascular disease, and osteoporosis), smoking history, diagnostic method, surgical method, operation time, and intraoperative blood loss were recorded for analysis. Location of dural tear was recorded by a chief surgeon as posterior, anterior, and lateral. Dural suturing was performed as far as possible and suture or non-suture repair was also recorded.

### Surgical method

Posterior surgery was performed to treat thoracolumbar disease. When dural was torn incidentally, a piece of cotton was placed above the gap to shelter the CSF leak, followed by the remaining procedures of decompression and/or fusion. According to the location of gap, the dural tear was sutured as far as possible. Finally, incision was closed by a conventional method in the CS group or fat graft combined with paraspinal muscle flap method in the FPM group.

### Conventional method

**Figure 1:**
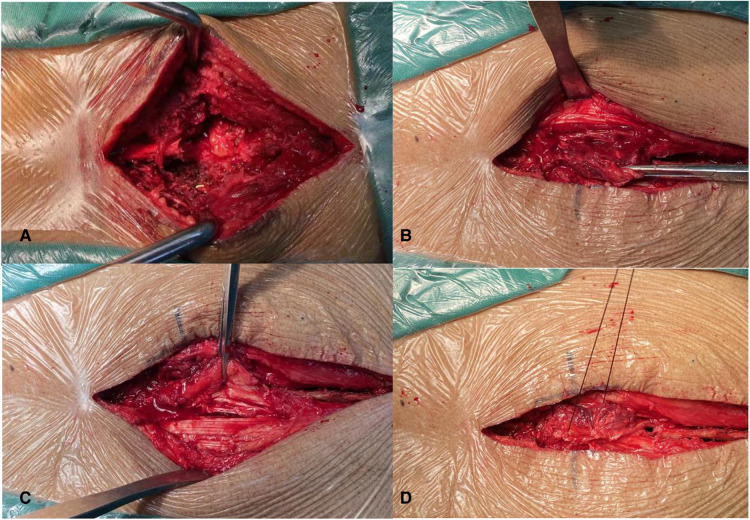
(**A**) A piece of autologous fat graft was used to shelter the dural gap; (**B–D**) paraspinal muscle flap was used for closing dead space of bilateral paraspinal muscles.

gelatin sponge was used to shelter the dural gap, the suturing was carried out layer by layer, and in particular, the layer of the thoracolumbar fascia should be tightly closed. Regarding the combination of fat graft with paraspinal muscle flap (Figure 1), autologous fat was removed from fat layer of sidewalls of incision that was used to shelter the dural gap (Figure 1A). Pedicle paraspinal muscle flap was made from both sidewalls of incision by tissue scissors. The location of pedicle paraspinal muscle flap was selected according to surrounding blood supply. Paraspinal muscle flap from a better blood supplied area was conducive to a longer survival. Two sides of paraspinal muscle flap were gathered and sutured together above the autologous fat for closing dead space of bilateral paraspinal muscles (Figure 1B). The suturing was performed in the same method as conventional method.

Drainage tube was placed routinely, and drainage pressure was normal and positive. Drainage tube time and total postoperative drainage volume were recorded in the two groups. Postoperative drainage was defined as the total volume from immediate post-operation to the time of tube removal.

### Clinical evaluation

Questionnaire is the main method to evaluate the clinical outcomes, and clinical evaluation was performed preoperatively, postoperatively (3 days after surgery), and in the last follow-up visit. Japanese Orthopedic Association (JOA) score was used to evaluate the neurological function. JOA score for patients undergoing lumbar spine surgery ranged from 0 to 29. For patients undergoing thoracic surgery, JOA score was modified and the score ranged from 0 to 11 (10).

In our study, the main clinical symptom of CSF leak was postural headache caused by the low intracranial pressure. Therefore, visual analogue scale (VAS) score was used to evaluate the headache preoperatively, postoperatively (3 days after surgery), and in the last follow-up visit in case of onset of symptoms. The VAS scoring was conducted as follows: a line was divided into 10 pieces (0 for no pain; 10 for the most severe pain), and patients used a pen to describe the degree of the pain in the line.

### Statistical analysis

In the present study, SPSS 22.0 software (IBM Corp., Armonk, NY, USA) was used to perform statistical analysis. *P* < 0.05 was considered statistically significant. According to the data distribution, the Student's *t*-test or the Mann–Whitney *U* test, as appropriate, was used for analysis of quantitative data in CS and FPM groups. The Chi-square test was used for analysis of qualitative data in CS and FPM groups. In each group, paired nonparametric test (Wilcoxon test) was utilized for comparing VAS score between postoperative and the last follow-up visit data. In each group, the paired *t*-test was used for comparing JOA score among preoperative, postoperative, and the last follow-up visit data. According to the Bonferroni correction, *P*-value was corrected to 0.0167 for decreasing type 1 error.

## Results

Table 1 compares patients’ basic characteristics between CS and FPM groups. There was no statistically significant difference in age (*P* = 0.561), gender (*P* = 0.609), BMI (*P* = 0.410), follow-up time (*P* = 0.878), medical comorbidities, and smoking history (*P* = 0.623) between the two groups.

**Table 1:** Comparison of patient basic characteristics between group CS and group FPM.

Variable	Total patients (*n* = 71)	Group CS (*n* = 34)	Group FPM (*n* = 37)	*P*-value
Age (years)	51.64 ± 11.92	50.76 ± 11.84	52.43 ± 12.10	0.561
Gender				0.609
Male	25	13	12	
Female	46	21	25	
Body mass index	25.31 ± 3.55	24.93 ± 3.42	25.64 ± 3.67	0.410
Follow-up period (m)	15.10 ± 4.15	15.18 ± 4.11	15.03 ± 4.24	0.878
Medical comorbidities				
Hypertension	26	13	13	0.786
Diabetes	14	5	9	0.309
Cardiac disease	17	9	8	0.632
Cerebrovascular disease	14	6	8	0.674
Osteoporosis	20	9	11	0.760
Smoking	21	11	10	0.623

Three thoracic spine-related diseases and three lumbar spine-related diseases were involved in the study (Table 2). Thoracic disc herniation, ossification of posterior longitudinal ligament, and ossification of ligamentum flavum were diagnosed in 1 patient, 3 patients, 9 and 2 patients, 3 patients, 12 patients in CS and FPM groups, separately. Lumbar disc herniation, spinal stenosis and spondylolisthesis were diagnosed in 3 patients, 15 patients, 3 and 3 patients, 11 patients, and 6 patients in CS and FPM groups, separately. The constituent ratio of diagnosis was not statistically different between the two groups (*P* = 0.813). Four surgical methods, including laminal decompression, laminal decompression with pedicle screws, laminal decompression with nucleus pulposus enucleation, and … were involved in the study, and there was no statistically significant difference between the two groups (*P* = 0.806, Table 2). The average operation time (*P* = 0.248) and intraoperative blood loss (*P* = 0.393) were similar in CS and FPM groups (Table 2).

**Table 2:** Diagnosis and surgical date in group CS and group FPM.

Variable	Group CS (*n* = 34)	Group FPM (*n* = 37)	*P*-value
Diagnosis			0.813
Thoracic	13	17	
Disc herniation	1	2	
Ossification of posterior longitudinal ligament	3	3	
Ossification of ligamentum flavum	9	12	
Lumbar	21	20	
Disc herniation	3	3	
Spinal stenosis	15	11	
Spondylolisthesis	3	6	
Surgical method			0.806
Lamina decompression	3	5	
Lamina decompression with pedicle screws	12	14	
Lamina decompression with nucleus pulposus enucleation	1	2	
Interbody fusion with pedicle screws	18	16	
Surgical time (min)	161.21 ± 47.62	174.86 ± 50.09	0.248
Intraoperative blood loss (ml)	314.85 ± 129.08	341.08 ± 125.78	0.393

Location of dural tear was recorded in posterior, anterior, and lateral areas of dura mater (Table 3). In the CS group, posterior CSF leak was detected in 23 patients, as well as anterior CSF leak in 8 patients, and lateral CSF leak in 3 patients, in which dural suturing was performed on 24 (70.59%) patients. In the FPM group, posterior, anterior, and lateral CSF leak were observed in 19, 10, and 8 patients, respectively, and dural suturing was carried out on 21 (56.76%) patients. No statistically significant difference was found in the location of dural tear (*P* = 0.252) and dural suturing (*P* = 0.227) between the two groups. The average drainage tube time in the FPM group was 3.89 ± 1.17 days, which was shorter (*P* < 0.001) than that (5.12 ± 1.56) in the CS group. The drainage volume in the FPM group (281.08 ± 284.76 ml) was also smaller (*P* < 0.001) than that in the CS group (859.70 ± 553.11 ml).

**Table 3:** Clinical date about CSF leakage in group CS and group FPM.

Variable	Group CS (*n* = 34)	Group FPM (*n* = 37)	*P*-value
Dural rupture location			0.252
Posterior	23	19	
Anterior	8	10	
Lateral	3	8	
Dural suture			0.227
Yes	24	21	
No	10	16	
Postoperative drainage tube time (days)	5.12 ± 1.56	3.89 ± 1.17	<0.001
Postoperative drainage (ml)	859.70 ± 553.11	281.08 ± 284.76	<0.001
Low intracranial pressure symptoms	15	7	0.022
Revision surgery	2	0	—

In the CS group, 15 (44.11%) patients complained of postural headache, and postoperative VAS score was 4.13 ± 1.36 that reduced to 0.67 ± 1.05 at the last follow-up visit (*P* = 0.001) (Table 4). Besides, 7 (18.91%) patients in the FPM group complained of postural headache, which was fewer than that in the CS group (*P* = 0.022). The postoperative VAS score in the CS group was 2.57 ± 0.98, and it decreased to 0.29 ± 0.49 at the last follow-up visit (*P* = 0.016) (Table 4). There was a statistically significant difference in postoperative VAS score (*P* = 0.013) between the two groups, however, no statistically significant difference was noted in the last follow-up visit VAS score between the two groups (*P* = 0.507). In both groups, postoperative and the last follow-up visit JOA scores were higher than those of preoperative JOA score (each *P* < 0.001); however, between the two groups, no statistically significant difference was found in preoperative JOA score (*P* = 0.912), postoperative JOA score (*P* = 0.595), and the last follow-up visit JOA score (*P* = 0.813) (Table 4). It is noteworthy that the incision in 1 patient in the CS group was not healed until 14 days after surgery, and CSF continued to flow out of the incision, therefore, revision surgery was performed. Delayed meningeal cyst was found in another patient in the CS group at 1 month after surgery, and subcutaneous cystic structure was touched around incision area. In addition, revision surgery was performed on patients who still complained of postural headache.

**Table 4:** Clinical outcomes in group CS and group FPM.

Variable	Group CS (*n* = 34)	Group FPM (*n* = 37)	*P*-value
VAS (headache)			
Postoperative	4.13 ± 1.36	2.57 ± 0.98	0.013
Last follow-up	0.67 ± 1.05*	0.29 ± 0.49*	0.507
*P*-value	0.001	0.016	
JOA			
Preoperative	12.03 ± 6.22	11.86 ± 6.25	0.912
Postoperative	16.06 ± 7.96*	15.05 ± 7.81*	0.595
Last follow-up	16.97 ± 7.76*	16.51 ± 8.27*	0.813

* Compared with preoperation, *P* < 0.005.

## Discussion

With the advent of an aging society, incidence of thoracolumbar disk disease is remarkably elevated. Thoracolumbar surgery is mainly complex, especially in the posterior approach, which is typically used to deal with complicated lesions or patients with a long course of disease. Ossification of the ligamentum flavum and calcification of the nucleus pulposus often result in adhesion to the dura, which can be easily damaged intraoperatively and lead to CSF leak. Thus, CSF leak is a common complication of thoracolumbar surgery that mainly occurs in the posterior approach (10–13). CSF leak was reported to occur in different incident rates in primary lumbar spine surgery versus revision surgery. Menon et al. (14) reported that the incidence of CSF leak in primary lumbar spine surgery ranged from 5.5% to 9%. Khan et al. (15) found that the incidence of dural tear in revision surgery was 15.9%, and this rate was 7.6% in primary lumbar spine surgery. Patients with CSF leak presented postural headache, continuous wound drainage, vertigo, posterior neck pain, etc. (1, 4). In the case of co-infection, meningitis may also occur, which is life-threatening (16). Tang et al. (16) demonstrated that timely dural repair greatly reduced the occurrence of complications and improved patients’ prognosis. However, in the large dural defect, the silk suture could not be used alone, and the suture itself also caused new dural holes, which increased the damaged dural area to some extent (8). Therefore, it is essential to clarify the method of dural repair and materials that could be used for dural repair, so as to improve patients’ prognosis.

A great number of methods were proposed to manage a dural tear. A study used bed rest only in the treatment of patients with CSF leak, and achieved satisfactory results (4). Hughes et al. (17) used Jackson-Pratt drainage to treat 8 patients with CSF leak, and those patients did not undergo dural repair. A promising prognosis was observed, all patients were discharged on time, and there was no complication associated with the long-term drainage tube (17). This might be related to the abundance of blood vessels in the dura, providing a strong ability to repair itself (18). Therefore, without surgical treatment, only conservative treatment is also effective for repairing dural tears. However, surgeons mainly preferred to repair the ruptured dura intraoperatively to achieve better results. Zhao et al. (19) also showed that the incidence of postoperative CSF leak was reduced by 5%–9% after intraoperative surgical treatment. Therefore, it is necessary to actively carry out intraoperative dural repair. Various materials can be used to repair dural tears, such as gelatin sponge, artificial dura, fibrin glue, fat graft, etc. (20). However, using different methods may cause different outcomes. In general, autologous fat/muscle/fascia tissue or gelatin sponge were mainly used for repairing dural tears (21). An autologous fat/muscle/fascia graft could be economic, with no rejection reaction, while it was difficult to achieve a tight waterproof (20, 22). A fat tissue can only play an anti-adhesion role in the early stage. The remaining fiber skeleton can be adhered to the surrounding tissue, forming a scar tissue when lipid droplets disappear at middle and late stages (23). However, a previous study (24) pointed out that the use of a fat graft caused complications of cranial fat dissemination. Therefore, some surgeons used gelatin sponges to repair the dural tears. As early as 1995, Narotam et al. (25) found that gelatin sponges promoted fibroblast activity, accelerated the dural regeneration, and had satisfactory clinical effects on preventing spinal nerve root adhesion. Tong et al. (26) demonstrated that hematopoietic stem and progenitor cells (HSPCs) could be enriched by implanting biomaterials into spatium intermusculare, which could be beneficial for patients’ recovery. However, Buyuktepe et al. (27) reported a case of acute hydrocephalus due to application of gelatin sponges, and it was revealed that CSF flow was immediately restored after removing this material, in which an additional ventriculostomy was performed. There is no clear conclusion to indicate which material is more appropriate for the treatment of CSF leak.

In our study, the method of using paraspinal muscle flap combined with fat graft for repairing CSF leak showed satisfactory clinical outcomes. Posterior dural tear was easy to suture, however, anterior and a part of lateral tear were sometimes difficult to suture. Intraoperative immediate suturing plays an important role in the reduction of the drainage volume. In the case of failure of dural suturing, more CSF could be leaked from the dura mater. Under this condition, paraspinal muscle flap combined with fat graft showed to be advantageous in decreasing postoperative drainage volume. There are viable adipose-derived stem cells, which might be differentiated into cellular structures required during dural repair (28, 29). In addition, bilateral paraspinal muscle flap eliminated the dead space of incision. Using these methods, postoperative drainage tube time or postoperative drainage volume could be significantly decreased. With the treatment of CSF leak, the incidence and the degree of postural headache were also significantly reduced. The method proposed in the current study showed promising results, particularly for the occurrence of CSF leak during posterior thoracolumbar surgery.

The present study evaluated the efficacy of utilizing fat graft combined with paraspinal muscle flap to treat CSF leak during posterior thoracolumbar surgery, however, there were several limitations that should be pointed out. First, the study was limited by its retrospective nature, and it was a single-center study with a small sample size. Second, no special biomaterials were included in the surgery. This could be related to the fact that there are numerous types of biomaterials for dural repairing, which could influence the accuracy of the surgery. Third, this study only included patients who underwent the primary lumbar spine surgery, and those who underwent the revision surgery were not involved. Factors influencing clinical outcomes in the revision surgery and the intraoperative situation should be explored. In addition, in the revision surgery, muscle tissue is replaced with scar tissue to varying degrees, thus, the proposed method is not always applicable.

## Conclusion

Fat graft combined with paraspinal muscle flap could be an effective method to treat CSF leak during posterior thoracolumbar surgery. The proposed method significantly reduced postoperative drainage tube time and postoperative drainage volume. It also decreased the incidence and the degree of postural headache. The proposed method showed satisfactory clinical outcomes, and it is worthy of promotion.

## Data Availability

The original contributions presented in the study are included in the article/Supplementary Material, further inquiries can be directed to the corresponding author/s.

## References

[1] Bosacco SJ Gardner MJ Guille JT. Evaluation and treatment of dural tears in lumbar spine surgery: a review. Clin Orthop Relat Res. (2001) 389:238–47. 10.1097/00003086-200108000-00033 11501817

[2] Schmidt D Setzer M Seifert V Marquardt G Bruder M. Resection of lumbar spinal facet joint cysts and cerebrospinal fluid leakage: incidence, prognostic parameters, and outcome in a single-center series. Clin Spine Surg. (2022) 5(6):E534–38. 10.1097/BSD.0000000000001309 35276717

[3] Sin AH Caldito G Smith D Rashidi M Willis B Nanda A. Predictive factors for dural tear and cerebrospinal fluid leakage in patients undergoing lumbar surgery. J Neurosurg Spine. (2006) 5(3):224–7. 10.3171/spi.2006.5.3.224 16961083

[4] Guerin P El Fegoun AB Obeid I Gille O Lelong L Luc S Incidental durotomy during spine surgery: incidence, management and complications. A retrospective review. Injury. (2012) 43(4):397–401. 10.1016/j.injury.2010.12.014 21251652

[5] Cammisa FP Jr. Girardi FP Sangani PK Parvataneni HK Cadag S Sandhu HS. Incidental durotomy in spine surgery. Spine. (2000) 25(20):2663–7. 10.1097/00007632-200010150-00019 11034653

[6] Stolke D Sollmann WP Seifert V. Intra- and postoperative complications in lumbar disc surgery. Spine. (1989) 14(1):56–9. 10.1097/00007632-198901000-00011 2913669

[7] Wang JC Bohlman HH Riew KD. Dural tears secondary to operations on the lumbar spine. Management and results after a two-year-minimum follow-up of eighty-eight patients. J Bone Joint Surg Am. (1998) 80(12):1728–32. 10.2106/00004623-199812000-00002 9875930

[8] Ito K Aoyama T Horiuchi T Hongo K. Utility of nonpenetrating titanium clips for dural closure during spinal surgery to prevent postoperative cerebrospinal fluid leakage. J Neurosurg Spine. (2015) 23(6):812–9. 10.3171/2015.3.SPINE141215 26315957

[9] Dafford EE Anderson PA. Comparison of dural repair techniques. Spine J. (2015) 15(5):1099–105. 10.1016/j.spinee.2013.06.044 23973097

[10] Sato T Kokubun S Tanaka Y Ishii Y. Thoracic myelopathy in the Japanese: epidemiological and clinical observations on the cases in miyagi prefecture. Tohoku J Exp Med. (1998) 184(1):1–11. 10.1620/tjem.184.1 9607393

[11] Wong AP Shih P Smith TR Slimack NP Dahdaleh NS Aoun SG Comparison of symptomatic cerebral spinal fluid leak between patients undergoing minimally invasive versus open lumbar foraminotomy, discectomy, or laminectomy. World Neurosurg. (2014) 81(3-4):634–40. 10.1016/j.wneu.2013.11.012 24239738

[12] Cho JY Chan CK Lee SH Choi WC Maeng DH Lee HY. Management of cerebrospinal fluid leakage after anterior decompression for ossification of posterior longitudinal ligament in the thoracic spine: the utilization of a volume-controlled pseudomeningocele. J Spinal Disord Tech. (2012) 25(4):E93–102. 10.1097/BSD.0b013e318246b89a 22425887

[13] Zhong J Wen B Chen Z. Predicting cerebrospinal fluid leakage prior to posterior circumferential decompression for the ossification of the posterior longitudinal ligament in the thoracic spine. Ann Palliat Med. (2021) 10(10):10450–8. 10.21037/apm-21-2323 34763491

[14] Menon SK Onyia CU. A short review on a complication of lumbar spine surgery: CSF leak. Clin Neurol Neurosurg. (2015) 139:248–51. 10.1016/j.clineuro.2015.10.013 26523872

[15] Khan MH Rihn J Steele G Davis R Donaldson WF 3rd Kang JD Postoperative management protocol for incidental dural tears during degenerative lumbar spine surgery: a review of 3,183 consecutive degenerative lumbar cases. Spine. (2006) 31(22):2609–13. 10.1097/01.brs.0000241066.55849.41 17047553

[16] Tang R Mao S Li D Ye H Zhang W. Treatment and outcomes of iatrogenic cerebrospinal fluid leak caused by different surgical procedures. World Neurosurg. (2020) 143:e667–e75. 10.1016/j.wneu.2020.08.069 32805467

[17] Hughes SA Ozgur BM German M Taylor WR. Prolonged Jackson-pratt drainage in the management of lumbar cerebrospinal fluid leaks. Surg Neurol. (2006) 65(4):410–4; discussion 4–5. 10.1016/j.surneu.2005.11.052 16531215

[18] Takai K Komori T Taniguchi M. Microvascular anatomy of spinal dural arteriovenous fistulas: arteriovenous connections and their relationships with the dura mater. J Neurosurg Spine. (2015) 23(4):526–33. 10.3171/2014.11.SPINE14786 26115024

[19] Fang Z Tian R Jia YT Xu TT Liu Y. Treatment of cerebrospinal fluid leak after spine surgery. Chin J Traumatol. (2017) 20(2):81–3. 10.1016/j.cjtee.2016.12.002 28336418 PMC5392710

[20] Sun X Sun C Liu X Liu Z Qi Q Guo Z The frequency and treatment of dural tears and cerebrospinal fluid leakage in 266 patients with thoracic myelopathy caused by ossification of the ligamentum flavum. Spine. (2012) 37(12):E702–7. 10.1097/BRS.0b013e31824586a8 22609726

[21] Lei T Shen Y Wang LF Cao JM Ding WY Ma QH. Cerebrospinal fluid leakage during anterior approach cervical spine surgery for severe ossification of the posterior longitudinal ligament: prevention and treatment. Orthop Surg. (2012) 4(4):247–52. 10.1111/os.12007 23109310 PMC6583446

[22] Yokota H Yokoyama K Nishioka T Iwasaki S. Active cerebrospinal fluid leakage after resolution of postdural puncture headache. J Anesth. (2012) 26(2):318–9. 10.1007/s00540-011-1291-4 22120169

[23] Görgülü A Simşek O Cobanoğlu S Imer M Parsak T. The effect of epidural free fat graft on the outcome of lumbar disc surgery. Neurosurg Rev. (2004) 27(3):181–4. 10.1007/s10143-003-0310-9 14534838

[24] Alhendawy I Tan D Homapour B. Cranial fat dissemination following fat grafting for lumbar dural tear: first case report in the literature. Int J Surg Case Rep. (2021) 81:105809. 10.1016/j.ijscr.2021.105809 33773369 PMC8024911

[25] Narotam PK van Dellen JR Bhoola KD. A clinicopathological study of collagen sponge as a dural graft in neurosurgery. J Neurosurg. (1995) 82(3):406–12. 10.3171/jns.1995.82.3.0406 7861218

[26] Tong JB Wu XY Jia GL Zhao KJ Wang SL Ma ZJ. Hematopoietic stem and progenitor cells can be enriched by implanting biomaterial into spatium intermusculare. Biomed Res Int. (2015) 2015:398642. 10.1155/2015/398642 25695072 PMC4324487

[27] Buyuktepe M Alpergin BC Abbasoglu B Orhan O Ozgural O. Acute hydrocephalus caused by a gelatin-sponge material: a case report. Childs Nerv Syst. (2022) 38(4):847–9. 10.1007/s00381-021-05292-8 34313829

[28] Rinker BD Vyas KS. Do stem cells have an effect when we fat graft? Ann Plast Surg. (2016) 76(Suppl 4):S359–63. 10.1097/SAP.0000000000000658 26545225

[29] Leberfinger AN Ravnic DJ Payne R Rizk E Hazard SW. Adipose-derived stem cells in peripheral nerve regeneration. Curr Surg Rep. (2017) 5(2):5. 10.1007/s40137-017-0169-2

